# Incidence, Risk, and Visual Outcomes after Repositioning of Acute Non-Traumatic Flap Dislocations Following Femtosecond-Assisted LASIK

**DOI:** 10.3390/jcm10112478

**Published:** 2021-06-03

**Authors:** Majid Moshirfar, David G. West, Chase M Miller, William B. West, Shannon E. McCabe, Kathryn M. Shmunes, Preston A. Baker, Yasmyne C. Ronquillo, Phillip C. Hoopes

**Affiliations:** 1 Hoopes Vision Research Center, Hoopes Vision, 11820 S. State Street Suite #200, Draper, UT 84020, USA; smccabe@hoopesvision.com (S.E.M.); yronquillo@hoopesvision.com (Y.C.R.); pch@hoopesvision.com (P.C.H.); 2 John A. Moran Eye Center, Department of Ophthalmology and Visual Sciences, University of Utah School of Medicine, Salt Lake City, UT 84132, USA; 3 Utah Lions Eye Bank, Murray, UT 84107, USA; 4 Brigham Young University, Provo, UT 84602, USA; gillison.west1@gmail.com; 5 McGovern Medical School at The University of Texas Health Science Center at Houston, Houston, TX 77030, USA; chase.m.miller@uth.tmc.edu (C.M.M.); Preston.A.Baker@uth.tmc.edu (P.A.B.); 6 School of Medicine, University of Utah, Salt Lake City, UT 84132, USA; William.West@hsc.utah.edu; 7 Mission Hills Eye Center, Pleasant Hill, CA 94520, USA; 8 Department of Ophthalmology, University of Florida College of Medicine-Jacksonville, Jacksonville, FL 32209, USA; kms04@gmail.com

**Keywords:** LASIK, corneal flap dislocation, FS-LASIK, corneal striae

## Abstract

Although the use of femtosecond lasers instead of mechanical devices has decreased the incidence of flap complications following laser-assisted in situ keratomileusis (LASIK), dislocations and striae still occur. Flap repositioning is an effective intervention to improve visual outcomes after acute flap complications in both microkeratome-assisted and femtosecond-assisted LASIK. This retrospective case series included patients undergoing flap repositioning secondary to acute flap dislocation and/or visually significant striae within the first two weeks following femtosecond LASIK (FS-LASIK) from 2015 to 2020 at a single institution. Preoperative, intraoperative, and postoperative de-identified data were analyzed for incidence, risk factors, and visual acuity outcomes. The incidence of flap repositioning was 0.35% in 21,536 eyes (*n* = 70). Indications for repositioning included acute flap dislocation (35.7%) and visually significant striae (64.3%). High myopia (OR = 3.04, *p* = 0.001) and patient age over 50 years (OR = 3.69, *p* = 0.001) were the strongest risk factors for these complications. Prior to flap repositioning, uncorrected distance visual acuity (UDVA) of 20/20 or better and 20/40 or better occurred in 19% and 57% of eyes, respectively. After repositioning, a final UDVA of 20/20 or better and 20/40 or better occurred in 78% and 98% of eyes, respectively. After repositioning, one line of UDVA was lost in two eyes (2.8%) and two lines were lost in one eye (1.4%). Risk factors for acute flap dislocation included high myopia and age over 50 years. Flap repositioning was effective in salvaging visual outcomes.

## 1. Introduction

Laser-assisted in situ keratomileusis (LASIK) is currently the most commonly performed refractive surgery in the United States, and visually significant complications are rare. Studies on the quality of life after LASIK surgery have shown that 96% of patients have an uncorrected distance visual acuity (UDVA) of 20/20 or better three months after surgery. Flap complications may require additional intervention to restore baseline corrected distance visual acuity (CDVA). The most common visually significant flap complications in the early postoperative period are striae and acute flap dislocation [1,2].

Technological advances in LASIK surgery, such as replacing the microkeratome with femtosecond lasers (FS-LASIK) for flap creation, have increased the precision of flap creation [3,4], increased flap adhesion, and reduced the incidence of flap-related complications [5]. However, acute flap dislocation and visually significant striae still arise at a rate of 1–2% [5,6,7]. In microkeratome-assisted LASIK, it has been established that a flap repositioning procedure is effective in salvaging preoperative visual acuity following acute flap dislocation and visually significant striae [2,6,7]. One study on microkeratome-assisted LASIK showed that patients undergoing flap repositioning (FR) procedures for striae have similar postoperative UDVA to eyes unaffected by striae [2]. Our study is a retrospective analysis of the incidence and outcomes of FR for acute flap dislocation and visually significant striae in patients undergoing FS-LASIK.

## 2. Patients and Methods

Retrospective chart review of all patients who underwent LASIK or enhancement of a prior LASIK procedure at Hoopes Vision between 1 January 2015 and 1 June 2020 was completed. Four different surgeons performed the LASIK procedures at a single site. The creation of the corneal flap was performed using the following femtosecond lasers based on surgeon preference: WaveLight FS200 (Alcon Laboratories, Inc., Fort Worth, TX, USA), Zeiss VisuMax (Carl Zeiss Meditec, Inc., Jena, Germany), or AMO iFS (Johnson & Johnson Vision, Santa Ana, CA, USA). This study involved de-identified patient data, adhered to the tenets of the Declaration of Helsinki, and was approved by the Hoopes Vision Ethics Review Board. The patients signed an informed consent for the use of their data in research prior to surgery. Brany IRB (New York, NY, USA) approved the exemption for this retrospective study.

Eyes requiring FR in the acute postoperative period were retrospectively identified. Only patients with a history of acute flap dislocation or visually significant striae who underwent FR within 14 days of the initial procedure were included in this study. Therefore, patients requiring FR secondary to post-LASIK trauma, interface debris, or diffuse lamellar keratitis (DLK) were excluded from this study. For patients who required bilateral FR, each eye was considered independently in data analysis. Indications for FR included visually significant microstriae, macrostriae, flap wrinkles, asymmetrical gutter, flap misalignment, flap dislocation, and flap dislodgement. Mechanical complications were classified into two groups: acute flap dislocation and striae. Slit lamp findings of flap wrinkles, asymmetrical gutter, misalignment, and dislodgement were all considered stages of flap dislocation when followed by FR procedures. Additionally, a large-volume control cohort composed of eyes undergoing LASIK from 1 January 2015 to 1 June 2020 at our facility without acute flap dislocations or striae was used for comparing preoperative and intraoperative risk factors. Some of the eyes in the control group did have other complications, including traumatic flap dislocation, epithelial ingrowth, and DLK. For age analysis, the control cohort was limited to patients aged 20 to 59 years.

### 2.1. LASIK Technique

The AMO iFS (Johnson & Johnson Vision, Santa Ana, CA, USA), WaveLight FS200 (Alcon Laboratories, Inc., Fort Worth, TX, USA), and Zeiss VisuMax (Carl Zeiss Meditec, Inc., Jena, Germany) femtosecond laser systems were used for flap creation, while the WaveLight EX500 excimer laser system (Alcon Laboratories, Inc., Fort Worth, TX, USA) was used for stromal ablation with a 6.0–6.5 mm central optical zone and blend zone to 8.5–9.0 mm. Flap diameter was between 8.5 and 9.0 mm, and flap thickness was between 100 and 115 µm with the creation of a superior hinge. The postoperative treatment protocol included ofloxacin 0.3% or moxifloxacin 0.5% four times a day for one week. Patients were instructed to apply prednisolone acetate 1% every 1–2 h while awake for the first 24 h. On the first postoperative day, the prednisolone was decreased to four times daily for one week and subsequently discontinued.

### 2.2. Flap Repositioning Technique

The flap edge was identified at the slit lamp using a Sinskey hook. The patient was taken to the operating microscope where they underwent draping with sterile technique. The flap was lifted and reflected superiorly with non-toothed forceps. A cellulose sponge was used to reflect any encroaching epithelium from the exposed stromal bed. Using a 27-gauge LASIK cannula, the flap was placed in the appropriate position and refloated to remove any debris and ensure proper alignment. For patients with significant macrostriae, the epithelium was debrided over the affected area. An 8.4 mm base curve bandage contact lens was placed after the procedure and then removed one week postoperatively. Patients were then strongly encouraged to avoid touching their eyes and to remain compliant with eye shield use.

### 2.3. Data Collection

Preoperative clinical data that were collected included the date of LASIK, age, sex, pre-LASIK medical and ophthalmic conditions, pre-LASIK systemic and ophthalmic medications, smoking status, pre-LASIK manifest refraction, UDVA, CDVA, uncorrected near visual acuity (UNVA), keratometry, and pachymetry. Age, sex, pre-LASIK UDVA, pre-LASIK CDVA, and manifest refraction were analyzed as possible risk factors for dislocation by comparison with the control cohort consisting of a random collection of eyes with no postoperative complications during the same time frame. Intraoperative parameters included femtosecond laser used, flap diameter, flap thickness, and surgery end time. Postoperative UDVA, CDVA, UNVA, and manifest refraction were recorded at one, three, six, and twelve months after LASIK.

Statistical analysis was performed using Microsoft Excel and R Statistics (version 3.5.0; Core Team 2018, Foundation for Statistical Computing, Vienna, Austria). The nonparametric Mann–Whitney test for independent samples was used to compare continuous outcomes. Fisher’s exact test and χ^2^ test were used to compare nominal outcomes when appropriate. A two-sided *p*-value of less than or equal to 0.05 was deemed statistically significant. Variables potentially associated with an increased likelihood of FR were investigated in a multivariable logistic regression. A *p*-value of less than or equal to 0.05 was deemed statistically significant. We used the standard nine graphs to report outcomes following refractive surgery.

## 3. Results

### 3.1. Incidence of Flap Repositioning Secondary to Acute Flap Dislocation and Striae

Out of 21,536 FS-LASIK procedures performed (including enhancements), 70 eyes required FR due to acute flap dislocation and striae for an overall incidence of 0.35%. The incidence of visually significant striae was 0.21%, and that of acute flap dislocation was 0.12%. The median age of the patients undergoing FR was 35 years (Table 1). At the time of intervention, 25 eyes (35.7%) had a flap dislocation, and 45 eyes (64.3%) had striae (Table 1). It is important to note that three patients required an enhancement procedure during the follow-up period after FR, and that these eyes were included in our analysis of VA. FR was performed one day after LASIK surgery in 91.4% of eyes (*n* = 64) (Table 1).

### 3.2. Preoperative and Intraoperative Characteristics of Patients Requiring Flap Repositioning

Table 1 shows the preoperative and demographic characteristics of our study group, while Table 2 compares these factors to a control group, randomly sampled from all eyes receiving LASIK between January 2015 and June 2020, for identification of possible risk factors for acute flap dislocation and visually significant striae requiring FR. The age at the time of the original procedure was significantly higher in FR eyes than in the control group (*p <* 0.001) (Table 2). The average sphere and spherical equivalent (SEQ) in the FR group were greater than in the control group (*p* < 0.001, *p* < 0.001 respectively), but the range of the sphere and SEQ was greater in the control cohort, indicating that eyes with greater degrees of myopia did undergo FS-LASIK surgery without complications (Table 2). Table 2 also shows stratified myopic patients based on the preoperative level of myopic sphere in diopters (D), showing that eyes with high myopia (greater than −6.0 D) had a greater incidence of FR than those with mild myopia (−0.50 to −1.50 D) to moderate myopia (−1.5 to −6.0 D) (*p* = 0.001). No other measured factors, including gender, laterality of affected eye, different femtosecond laser used, performing surgeon, preoperative keratometry, or diopters of cylinder, showed a statistically significant difference between the FR group and the control group.

Table 3 calculated the odds ratios of characteristics measured to have statistically significant differences from the control group in Table 2. Eyes with high myopia were shown to have a significantly greater risk of FR (OR = 3.04, 95% CI = 1.56–6.14, *p* = 0.001) (Table 3). Patients 50 years and older were also shown to have a statistically significant increase in the risk of needing FR (OR = 3.69, CI 95% = 1.57–7.93, *p* = 0.001) (Table 3).

### 3.3. Visual Outcomes of Flap Repositioning Patients

**Figure 1:**
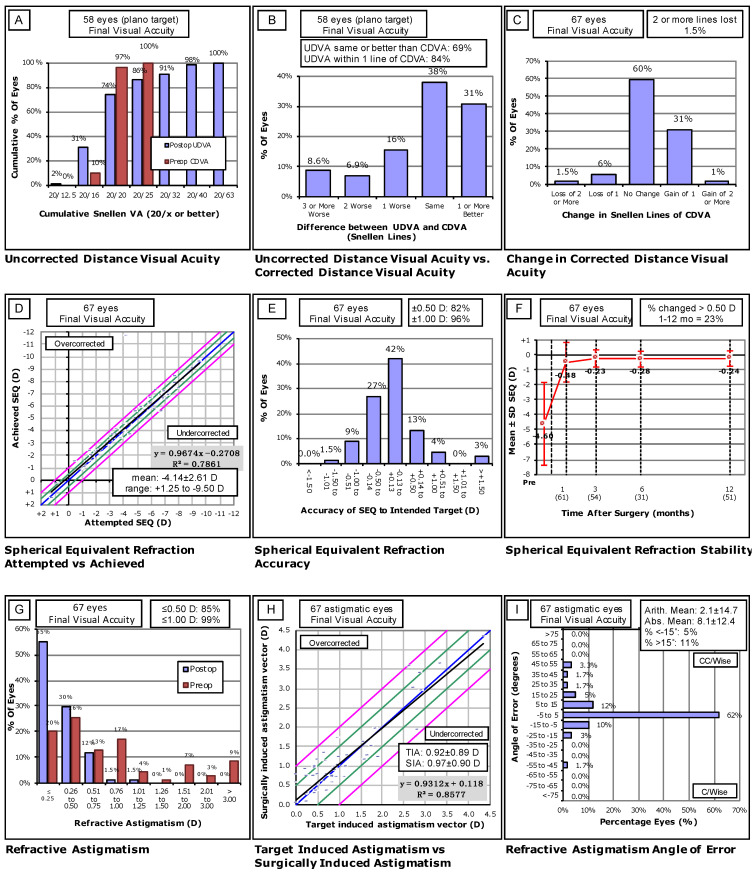
Standard nine graphs for reporting outcomes following refractive surgery. (**A**) Proportion of plano targeted eyes achieving a certain UDVA following repositioning compared to CDVA pre-LASIK. (**B**) Difference in Snellen lines between pre-LASIK CDVA and post-repositioning UDVA for plano targeted eyes (**C**) Difference in Snellen lines between pre-LASIK and post-repositioning CDVA. (**D**) Plot showing the association between final achieved SEQ following repositioning compared to pre-LASIK attempted SEQ. The blue line indicates attempted = achieved. Green lines indicate +/− 0.50 D. Pink Lines indicate +/− 1.00 D. (**E**) Accuracy of final post-repositioning SEQ compared to pre-LASIK target SEQ (**F**) Stability of mean SEQ over the follow-up period following repositioning. (**G**) Proportion of eyes achieving a certain astigmatism (D) before and after LASIK and repositioning procedures. (**H**) Plot showing the association between target induced astigmatism (TIA) and surgically induced astigmatism (SIA) following repositioning compared to pre-LASIK TIA vector. The blue line indicates attempted = achieved. Green lines indicate +/− 0.50 D. Pink lines indicate +/− 1.00 D. (**I**) Final angle of error of astigmatism post-repositioning SIA compared to pre-LASIK TIA.

Preoperative CDVA of 20/20 or better was seen in 98% of eyes. At diagnosis of flap dislocation, 16% of eyes had an UDVA of 20/20 or better, with 53% having an UDVA of 20/40 or better. One month following FR, 67% of eyes had an UDVA of 20/20 or better, with 100% having an UDVA of 20/40 or better. Three months following FR, 65% of eyes had an UDVA of 20/20 or better, with 96% having an UDVA of 20/40 or better. A final UDVA of 20/20 or better and 20/40 or better was reached within 12 months in 74% and 98% of FR eyes with a plano target, respectively (Figure 1A). SEQ within 0.50 D of the target pre-LASIK SEQ was achieved in 82% of eyes, and 96% were within one diopter of the pre-LASIK target SEQ (Figure 1E). Surgically induced astigmatism (SIA) within 15 degrees of target induced astigmatism (TIA) was reached in 84% of eyes, with variance in astigmatism being greater preoperatively than postoperatively (Figure 1I). These results are significant, as 74.3% of eyes in the study cohort were noted to have astigmatism preoperatively (Table 2). One patient with high myopia (SEQ = −11.75 D OD and −12.125 D OS) received a planned implantable collamer lens (ICL) during the follow-up period as a supplement to FS-LASIK. To account for the ICL, this patient’s target SEQ was significantly more myopic than all other eyes in the cohort (Figure 1D). In this patient, the difference from the intended target astigmatism was <0.50 D in both eyes. The final UDVA in this patient was 20/40 OD and 20/25 OS, and the final CDVA was 20/25 OD and 20/20 OS.

## 4. Discussion

This study adds to the literature on the visual outcomes of eyes undergoing a flap repositioning procedure for acute flap dislocation and striae secondary to FS-LASIK [3,4,5,8,9,10,11]. Incidence of repositioning (0.33%), visually significant striae (0.21%), and flap dislocation (0.12%) was within the reported range for FS-LASIK procedures based on data reported by the American Academy of Ophthalmology (0.01–0.40% for flap dislocation, 0.6% for striae) [3,12]. In this study, the incidence of FR was less than half of that measured in a similar study evaluating LASIK using a microkeratome (0.79%) [2]. This is consistent with other studies comparing microkeratome and FS-LASIK, in which femtosecond laser was shown to decrease the rate of acute flap dislocation and striae compared to microkeratome [4,5]. Clare et al. reported that early displacement (within 48 h) of the flap after LASIK occurs in approximately 1–2% of cases [5].

It is worth noting that the criteria for diagnosis and classification of flap dislocation and striae can vary widely among clinicians. We included asymmetrical gutter, flap misalignment, flap dislocation, and flap dislodgement, but other studies have additionally included buttonholes, torn flaps, and flap dislocations outside of the time period measured in this study (most commonly, traumatic flap dislocations) [3]. There are multiple possible explanations for the large variation in the incidence of acute flap dislocation and striae among different studies. These include differences in surgeon experience, choice of wavefront custom laser ablation versus conventional laser ablation, and variation between sample populations, such as the preoperative characteristics reported in Table 1 [2,4,5,6].

In this study, there was no significant difference in the incidence of acute flap dislocation or striae from year to year. In contrast, Mimouni et al. described inter-surgeon variability in complication rates and decreasing complication rates year to year as they performed more FS-LASIK procedures [6].

In our population, there was also no statistical difference in dislocation rates between surgeons or between different femtosecond laser models (Table 2). This is interesting in that the surgeons and lasers in our study used different default flap sizes, yet still showed no difference in dislocation rates. In contrast, past studies on FS-LASIK showed that increased flap size was associated with higher dislocation rates. 

High myopia is an established risk factor for acute flap dislocation, although dislocations remain rare even among high myopes [2]. Our data also showed that acute flap dislocations and striae are more likely to occur in myopic eyes (Table 3). It has been proposed that the increased risk of dislocation in these patients could be due to a reduction in postoperative stromal bed thickness. This is supported by several studies that found an increased risk of acute flap dislocation in patients with higher total stromal ablation percentages [2,13]. Increased precision of flaps created with femtosecond lasers could allow surgeons to minimize the total percent ablation in patients with at-risk corneas, including high myopes.

Patients 50 years and older were significantly more likely to suffer acute flap dislocations (Table 3). Previous studies have demonstrated the impact of age on the visual outcomes of refractive surgery [14,15,16,17]. However, to our knowledge, no previous study has shown increased age as a preoperative risk factor for acute flap dislocation following FS-LASIK. Increased risk of flap dislocation in older patients may be due to age-related changes in the cornea. Senescent changes of the cornea include a reduction in keratocyte and endothelial cell densities [18,19,20], corneal stiffening [21,22], epithelial barrier dysfunction [23,24,25,26], and a greater incidence of dry eye [27,28,29,30]. We postulate that these and other age-related changes may lead to slower corneal healing and decreased flap adhesion, resulting in a greater incidence of flap dislocation.

The correlation between the incidence of dry eye and age is well documented in the literature [27,28,29,30]. Dry eye may reduce lubrication between the eyelid and the corneal flap, possibly leading to an increased risk of flap dislocation. Dry eye may also contribute to the difference in the incidence of flap dislocation between FS-LASIK and LASIK with a microkeratome, as FS-LASIK has a significantly lower incidence of dry eye [31].

Changes in the ability of the flap to adhere may also contribute to flap dislocation. Keratocytes play a crucial role in corneal wound healing by secreting growth factors and producing collagen [32,33,34,35]. The reduction in keratocyte density seen with aging likely contributes to slower flap healing in older patients [18,19,20]. Theories for the mechanism of early flap adhesion include osmotic pressure due to endothelial pump activity [36,37], electrostatic interactions in the stroma [38], and epithelial bridging at the edge of the flap [37]. Multiple studies have found brimonidine to reduce flap adherence and increase the risk of dislocation and cite its effects on endothelial pump activity as a possible cause [39,40,41]. Endothelial cell density and endothelial pump activity both decrease with age and could be related to an increased incidence of flap dislocation in older patients if osmotic pressure is indeed the mechanism for early flap adhesion [18,19,20,42]. However, no conclusive evidence for any of the possible mechanisms of flap adhesion was found in our review of current literature. Further investigation into the mechanism of flap adhesion following FS-LASIK is warranted to better understand why age increases the risk of acute flap dislocation and to suggest possible interventions to mitigate risk.

Flap repositioning procedures following FS-LASIK successfully salvaged CDVA equal to or better than that measured preoperatively in 94.5% of patients suffering from acute flap dislocation or striae. This rate is similar to that seen in a study on LASIK using a microkeratome, in which a 92.6% safety index was achieved [2].

In our study, four eyes (5.7%) lost one or more Snellen lines of CDVA, with one eye losing two lines of CDVA and three eyes losing one line of CDVA. Of these four eyes, two had a preoperative CDVA of 20/15 with a postoperative CDVA of 20/20. However, it is important to note that the examiner may not always check beyond the 20/20 Snellen line, which could account for the apparent reduction in CDVA in eyes with a preoperative CDVA of 20/15. It is also important to note that a large angle of error of refractive astigmatism was found in three out of four of these eyes, which could account for the reduction in perceived CDVA. Overall, we conclude that patients suffering acute flap dislocation or visually significant striae following FS-LASIK are unlikely to lose visual acuity if the flap is repositioned.

In our study, 69% of eyes undergoing FR had an efficacious outcome, defined as final UDVA equal to or better than pre-LASIK CDVA (Figure 1B). Our results are similar to Wallerstein et al.’s study on microkeratome-assisted LASIK, where an efficacious outcome was achieved in 70.2% of eyes [2]. Based on the results shown in Figure 1G,H, patients with astigmatism who suffer acute flap dislocation and striae treated by FR do not appear to have inferior outcomes to those who do not have astigmatism.

The external validity of this study may be limited due to the data collection occurring at only one center and possible inter-patient variability between study groups. Another limitation in our study is that 27% of our patients were lost to follow-up before reaching the 12-month postoperative mark. Further directions include performing a multivariate analysis of total ablation depth and percent tissue altered in FS-LASIK as potential risk factors for flap dislocation. 

## 5. Conclusions

Flap repositioning led to satisfactory visual outcomes in cases of acute non-traumatic flap dislocation following FS-LASIK. Significant risk factors for acute dislocation were high myopia (greater than −6.0 D) and age over 50 years. No other preoperative, intraoperative, or postoperative risk factors were associated with increased risk of acute flap dislocation.

## Figures and Tables

**Table 1:** Demographics and risk factors of patients undergoing flap repositioning following LASIK.

Demographic/Risk Factor	Median (IQR)	Range
Age (Years)	35 (14)	20–61
	*n* (eyes)	%
Requiring Repositioning Following First LASIK	68	97.10%
Requiring Repositioning Following Enhancement	2	2.90%
Gender (Male/Female)	31/39	44.3%/55.7%
Affected Eye		
OD Only	28	40%
OS Only	38	54.30%
OU	4	5.70%
Refractive Error		
Simple Myopia	8	11.40%
Simple Hyperopia	0	0.00%
Compound Myopic Astigmatism	60	85.70%
Compound Hyperopic Astigmatism	2	2.80%
Indications for Repositioning		
Flap Dislocation	25	35.70%
Striae	45	64.30%
Postoperative Days to Repositioning		
1 Day	64	91.40%
2–7 Days	1	1.40%
8–14 Days	5	7.10%

IQR: Interquartile Range; OD: right eye; OS: middle eye; OU: both eyes.

**Table 2:** Preoperative and intraoperative variable comparison in repositioned eyes vs. control cohort.

	Repositioning Group (*n* = 70)		Control Cohort (*n* = 14,418)		
Variable	Mean ± SD	Range	Mean ± SD	Range	*p*-Value
Age	35 ± 8.50	20, 61	32 ± 9.93	17, 85	**0.001**
Keratometry					
Kf	43.35 ± 1.58	37.6, 47.5	43.281 ± 1.51	31.2, 46.9	0.6926
Ks	44.54 ± 1.70	38.7, 48.1	44.5 ± 1.61	31.9, 49.0	0.8147
Km	43.94 ± 1.56	38.1, 47.7	43.89 ± 1.50	31.55, 47.8	0.8045
Manifest Refraction					
Sphere (D)	−4.11 ± 2.68	−10.0, +3.00	−3.03 ± 2.21	−12.0, +6.25	<0.001
Cylinder (D)	−1.00 ± 1.02	−4.50, 0	−0.93 ± 0.96	−7.50, +2.25	0.4822
Spherical Equivalent (D)	−4.62 ± 2.72	−12.1, +0.88	−3.49 ± 2.15	−13.2, +4.38	<0.001
	*n*	Percent	*n*	Percent	*p*-value
Gender	31/39	44.3%/55.7%	7440/6978	51.6%/48.4%	0.27
Refractive Error					0.696
Simple Myopia	8	11.40%	2081	[14.5%]	
Simple Hyperopia	0	0.00%	47	[0.3%]	
Compound Myopic	60	85.70%	11,594	[80.6%]	
Astigmatism					
Compound Hyperopic	2	2.80%	659	[4.6%]	
Astigmatism					
Laser					0.615
AMO iFS	59	[84.3%]	12,014	[83.5%]	
WL FS200	5	[7.1%]	843	[5.9%]	
Zeiss VisuMax	6	[8.6%]	1524	[10.6%]	
Surgeon					0.728
A	59	[84.3%]	11,716	[81.5%]	
B	6	[8.6%]	1221	[8.5%]	
C	4	[5.7%]	794	[5.5%]	
D	1	[1.4%]	650	[4.5%]	

Significant *p* value in bold.

**Table 3:** Preoperative risk factors for flap repositioning.

	Sphere (D)	Eyes (*n*)	% Requiring	Flap	Odds Ratio	95% CI	*p*-Value
			Reposition	Reposition (*n*)			
Age							
≤29 *		4657	0.43%	20	-	-	-
30–39		6147	0.41%	24	0.9	0.49–1.64	0.717
40–49		2297	0.61%	14	1.4	0.69–2.76	0.336
≥50		638	1.41%	9	3.69	1.57–7.93	**0.001**
Myopia Severity							
Mild *	−0.50 to −1.50	3026	0.465	14	-	-	-
Moderate	−1.5 to −6.00	8828	0.34%	30	0.74	0.39–1.45	0.365
Severe	≥−6.00	1677	1.37%	23	3.04	1.56–6.14	**0.001**

* Used as baseline data for category; Significant *p*-value in bold.
